# Database of SARS-CoV-2 and coronaviruses kinetics relevant for assessing persistence in food processing plants

**DOI:** 10.1038/s41597-022-01763-y

**Published:** 2022-10-26

**Authors:** Ngoc-Du Martin Luong, Laurent Guillier, Sandra Martin-Latil, Christophe Batejat, India Leclercq, Christine Druesne, Moez Sanaa, Estelle Chaix

**Affiliations:** 1 Risk Assessment Department, ANSES, Maisons-Alfort, France; 2 Laboratory for Food Safety, ANSES, University of Paris-EST, Maisons-Alfort, France; 3 Institut Pasteur, Université Paris Cité, Environment and Infectious Risks Unit, Laboratory for Urgent Response to Biological Threats (CIBU), Paris, France; 4 Research fundings & scientific watch department, ANSES, Maisons-Alfort, France

**Keywords:** Virology, Computational biology and bioinformatics

## Abstract

SARS-CoV-2 (Severe acute respiratory syndrome coronavirus 2), a virus causing severe acute respiratory disease in humans, emerged in late 2019. This respiratory virus can spread via aerosols, fomites, contaminated hands or surfaces as for other coronaviruses. Studying their persistence under different environmental conditions represents a key step for better understanding the virus transmission. This work aimed to present a reproducible procedure for collecting data of stability and inactivation kinetics from the scientific literature. The aim was to identify data useful for characterizing the persistence of viruses in the food production plants. As a result, a large dataset related to persistence on matrices or in liquid media under different environmental conditions is presented. This procedure, combining bibliographic survey, data digitalization techniques and predictive microbiological modelling, identified 65 research articles providing 455 coronaviruses kinetics. A ranking step as well as a technical validation with a Gage Repeatability & Reproducibility process were performed to check the quality of the kinetics. All data were deposited in public repositories for future uses by other researchers.

## Abstract (summary)

**Table:** 

Measurement(s)	Decimal reduction time
Technology Type(s)	Modelling of kinetics
Factor Type(s)	Temperature • Relative humidity • Genus • Strain
Sample Characteristic - Organism	Coronaviridae

## Background & Summary

The first cases of coronaviruses disease 2019 (COVID-19) due to SARS-CoV-2 were detected in China in December 2019 and spread afterwards quickly around different countries from around January-February 2020^1^. From the first months of viral dissemination, SARS-CoV-2 clusters were observed, in particular in occupational environments for several essential sectors such as health care centres^2,3^ or food processing plants^4–7^. The transmission of coronaviruses among humans was reported as possibly through aerosol (inhalation of aerosolized or falling contaminated droplets) or through contact (hand, objects or surfaces)^8,9^. Infected persons (symptomatic or asymptomatic) can send out several contaminated droplets that could stay in aerosol, be directly inhaled or fall on surfaces and potentially infect afterwards other persons. However, the changes of the infectious viral load in the contaminated droplets susceptible to be inhaled and to induce disease are not well known in either aerosol or surfaces. Furthermore, the persistence of SARS-CoV-2 depends on environmental conditions that are very different from one occupational location to another^10,11^. Some factors such as airflow, ventilation, temperature and relative humidity modify the probability of SARS-CoV-2 transmission through respiratory pathways since it can affect droplet movements and virus survival, notably through droplet desiccation^12^. Some studies reported the influence of few temperature and/or relative humidity conditions. For example, the virus remains infectious for longer periods at lower temperatures and very high relative humidity^10^, metal surfaces such as stainless steel could allow the virus to remain infectious longer than on others under some specific temperature-humidity conditions^13,14^. Studies on coronaviruses persistence were generally conducted by experiments in laboratory consisting in monitoring the virus kinetics under controlled conditions. The reduction of virus infectivity over time could be evaluated by fitting inactivation mathematical models on experimental data. Studying the effects of environmental conditions encountered in food premises requires collecting kinetics data as exhaustively as possible to cover large ranges of values for each condition: inert or food surfaces, temperature, relative humidity, experimental quantification method, virus strain etc. To our knowledge, such exhaustive kinetics dataset are not available yet in the literature. It should also be noted that the gathering of data between different studies is not easy. For example, kinetics are frequently plotted in research articles but raw data are not often provided in all of them as quantitative (numerical) values that can be used for further studies of theirs.

Thus, the main goal of this paper was to collect and make available the compilation of a large dataset of quantitative kinetics related to SARS-CoV-2 and other coronaviruses under different conditions useful for assessing and modelling their persistence in food processing environments.

## Methods

**Fig. 1:**
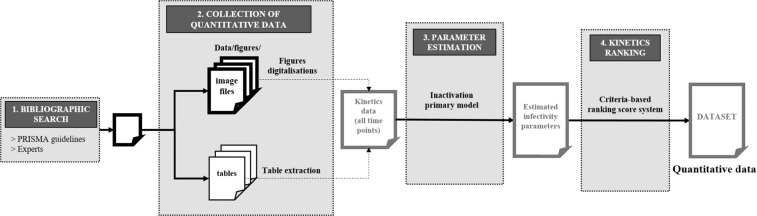
Schematic overview of the data collection workflow.

The overall procedure for collecting and pre-treating literature data is briefly illustrated in Fig. 1. Firstly, a literature review to identify relevant publications presenting kinetics data was carried out. This research was based on a query of scientific bibliographical databases in accordance with the PRISMA guidelines^15^ (Step 1). The second step consisted in converting raw data from scientific publications (texts, tables or figures) into a ready-to-use numerical dataset with a manual collection for tables or a semi-automated collection after digitalization of figures (Step 2). Afterwards, an inactivation primary model was fitted on each kinetics to estimate the viral infectivity reduction parameter and its uncertainty (Step 3). Finally, a quality-ranking step (Step 4) was performed to evaluate the quality of the data collected from kinetics. The quantitative dataset and the different tools used in this procedure are freely available and detailed in the following sub-sections.

### Scoping review

**Fig. 2:**
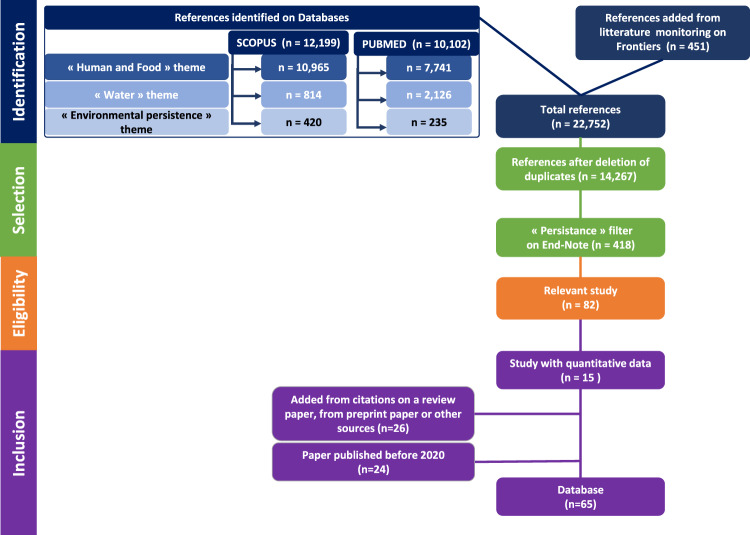
Flow chart outlining the procedure for quantitative data collection from the literature based on the PRISMA guidelines statement and preliminary studies.

The scoping review is part of a scientific project to describe the persistence of coronaviruses in food production environments. Firstly, a query process was carried out to identify relevant records, using weekly advanced searches on several topics associated with SARS-CoV-2. This weekly literature search was conducted between March 2020 and 25^th^ August 2021 using a combination of keywords related to the main thematic (1) “SARS-CoV-2 and coronavirus” joined by the logical connector AND with one of the following (2a) “Human and food”, (2b) “Water” or (2c) “Environmental persistence”. The keywords used for each theme are specified in Table 1. Studies were collected weekly from two bibliographic search engines PubMed and Scopus (from March 2020 to August 2021) and by query on Frontiers (from November 2020 to August 2021). A date restriction was defined: only publications from the 1^st^ of January 2020 were collected. The search was limited to publications with abstracts written in English. The last query process using those above-mentioned criteria performed on the 25^th^ of August 2021 identified overall 14,267 references exported to EndNote software, after duplicate removal. From this corpus, a thematic filter about “Persistence” was built with a “from group” tools in EndNote software. This filter consisted in selecting articles with “persisten*” “survival” or “stability” in the field “Title–Abstract–Keywords”, joined by the logical connector AND with “environment*”. This search resulted in 418 references. These records were afterwards filtered in accordance with the PRISMA Statement guidelines^15^ (Fig. 2), using different inclusion/exclusion criteria based on title, abstract and sometimes full-text when needed. The inclusion criteria were (1) studies on persistence on materials, surfaces or aerosols or (2) studies on working environment. The exclusion criteria were (1) studies on therapeutic or vaccine development, (2) studies on untreated wastewater, (3) studies on diet or nutrition (4) language other than English and French, and (5) full text not available. A first screening step identified 82 references for which full papers were read to determine if they were included as fully documented kinetics of persistence data (either in tables or in figures) or used to complete the identification of relevant publications (e.g. in the case of reviews). All the above screening and completion stages identified a final total number of 65 studies in which available raw kinetics data could be extracted from tables and/or figures.

**Table 1:** Keywords used in the four themes for weekly bibliographic database queries.

Query theme	*Keywords*
(1) “SARS-CoV-2 and coronavirus”	*“sars-cov-2”* OR *“covid-19”* OR “*coronavirus”* OR “*corona virus”* OR *“2019-ncov”* OR *“novel coronavirus”*
(2a) “Human and food”	“*food” OR “bread”* OR “d*airy products” OR “eggs”* OR *“fast foods” OR “flour”* OR “*fruit”* OR “*meal”* OR “*meat”* OR “*raw foods”* OR “*salads”* OR “*vegetables” OR “foodborne”* OR “*gastrointestinal”* OR “*intestine”* OR “*digestive”* OR “*feces”* OR “*stool”* OR “*fecal” OR “clams”* OR “*oysters”* OR “*cockles”* OR “*mussels”* OR “*scallops”* OR “*molluscs”* OR “*bivalvia”* OR “*shellfish fish” OR “shellfish farming”*
(2b) “Water”	“*seawater” OR “sea water”* OR “*marine”* OR “*wastewater” OR “water treatment plant”* OR “water”
(2c) “Environmental persistence”	“environment*” AND (“*survival” OR “persistence”*)

### Information associated with kinetics data

The second step consisted in converting raw data from scientific publications into ready-to-use numerical dataset by extracting data from texts, tables and/or using figure digitalization techniques. This step provided several “kinetics”, meaning the tracking of viral titer at different time points under different conditions. Kinetics corresponding to viral genome quantification (e.g. by RT-qPCR techniques) were excluded since such quantification did not represent the viral infectivity. In total, 464 kinetics were available from the 65 identified studies^11,13,14,16–77^ (Tables 2 and 3). It is worth noting that kinetics for coronaviruses other than SARS-CoV-2 collected in these studies were also retained for analyses.

**Table 2:** Overview of the persistence kinetics of SARS-CoV-2 on different matrices and the associated environmental conditions.

Standardised/Classified matrix	Temperature (°C) *	Relative humidity (%) *	Number of kinetics	References
Dechlorinated tap water, autoclaved wastewater	4, 15, 25, 37	100	9	^38^
Biological specimen, Liquid culture medium	56	100	3	^27^
Biological specimen	65			
Coated glass, Stainless, Glass	22	65	16	^39^
Stainless steel	24, 35	20, 40, 60	6	^40^
Glass, Liquid culture medium	4, 22.5, 30, 37	63, 100	8	^45^
Liquid culture medium	4, 22, 37, 56	100	14	^14^
Cotton, Glass, Mask, Paper, Plastic, Stainless steel, Wood	22	65		
Food (salmon)	4, 25	N/A	4	^46^
Liquid culture medium		100		
Biological specimen, Food (Dairy drinks; Juice/Coffee/Tea; Alcohol/Carbonated/Health beverage), Liquid culture medium, Water	4	100	26	^48^
Glass, Plastic, Stainless steel	22	40	2	^90^
Skin	4	45	12	^50^
Liquid culture medium	4	100	6	^51^
Cotton, Latex, Mask, Plastic, Rubber, Stainless steel, Tyvek	20	37.5	8	^52^
Metal	4, 22, 30	35	3	^53^
Cardboard, Cloth, Concrete, Foam, Glass, Mask, Polyproylene, Rubber, Stainless steel, Tyvek	21	60, 66, 70, 75	48	^54^
Water	23	100	4	^55^
Ceramic, Glass, Mask, Latex, Cotton, Paper, Plastic, Stainless steel, Wood	20	N/A	9	^56^
Plastic	22.5, 28	40	2	^58^
Biological specimen	4, 21	40	6	^59^
	27	85		
Food (Dairy drinks)	5	100	1	^91^
Paper, Plastic	21.8	42.8	5	^61^
Plastic	21.9	37.4	5	^62^
Paper, Plastic, Foam	21.8	38.6	5	^63^
Leather	22	38.6	2	^64^
Brass, Glass, Marble, Plastic, Stainless steel	21,7	36.6	5	^65^
Foam, Paper, Plastic	2.4	34.5	8	^66^
	28.6	32.6		
Aluminum, Glass, Plastic	20	50	6	^68^
Cotton, Mask, Plastic, Polymer note, Polyester, Stainless steel, Tyvek	21.5	45	7	^69^
Stainless steel	19	57	3	^70^
Cotton, Glass, Paper, Polymer note, Stainless steel, Vinyl	20, 30, 40	50	18	^71^
Acrylic, Laminate, Plastic, Polyurethane, Quartz, Rubber, Vinyl, Stainless steel	25	47.5	14	^72^
Water	20	100	1	^41^
Water	4, 20	100	4	^77^
Aerosol in liquid culture medium	20.5	78	4	^75^
Aerosol, Cardboard, Copper, Plastic, Stainless steel	22	40	5	^13^
Stainless steel	7, 25	65	4	^76^
Plastic	20, 22, 27	40, 65, 85	9	^11^

*median values were considered if temperatures and relative humidity were given by ranges.

**Table 3:** Overview of the persistence kinetics of coronaviruses (*Alphacoronavirus* and *Betacoronavirus* other than SARS-CoV-2) on different matrices and the associated environmental conditions.

Genus	Sub-genus	Virus	Standardized/Classified matrix	Temperature (°C) *	Relative humidity (%) *	Number of kinetics	Reference
*Alphacoronavirus*	*Duvinacovirus*	HCoV 229E	Cotton, Polycotton, Polyester,	19	34	3	^67^
		HCoV 229E	Liquid culture medium, Plastic	23, 56, 23	100	4	^18^
		HCoV 229E	Stainless steel	7, 25	65	4	^76^
		HCoV 229E	Food (vegetables)	22	35	4	^42^
		HCoV 229E	Glass, Plastic, Stainless steel	24	50	3	^43^
		HCoV 229E	Liquid culture medium	33, 37	100	4	^22^
		HCoV 229E	Liquid culture medium	4, 22, 33, 37	100	4	^32^
		HCoV 229E	Ceramic, Glass, PVC, Rubber, Stainless, Teflon	21	35	6	^26^
		HCoV 229E	Liquid culture medium	37	100	3	^74^
		HCoV 229E	Water	23	100	1	^30^
	*Pedacovirus*	PEDV	Liquid culture medium	50	100	1	^33^
		PEDV	Liquid culture medium	48	100	9	^35^
		PEDV	Liquid culture medium	40, 44, 48	100	9	^34^
	*Tegacovirus*	Alphacoronavirus 1	Plastic	4	N/A	3	^57^
		FCoV	Stainless steel	7, 25	65	4	^76^
		FIPV	Liquid culture medium	54	100	1	^28^
		FIPV ATCC-990	Water	23	100	1	^30^
		PRCV	Liquid culture medium, Seawater, Water	20	100	3	^47^
		TGEV	Lake water, Reagent-grade water	25	100	2	^21^
		TGEV	Stainless steel	4, 20, 40	20, 50, 80	8	^20^
		TGEV	Cotton, Latex, Mask, Nitrile glove	20	50	4	^44^
		TGEV	Liquid culture medium	31, 35, 39, 43, 47, 51, 55	100	12	^19^
*Betacoronavirus*	*Embecovirus*	BCoV	Food (vegetables), Liquid culture medium	4	100	2	^37^
		CCV	Liquid culture medium	60	100	1	^73^
		HCoV OC43	Cotton, Polycotton, Polyester	19	34	3	^67^
		HCoV OC43	Liquid culture medium	33, 37	100	4	^22^
		HCoV OC43	Liquid culture medium	37	100	3	^74^
		MHV	Liquid culture medium	40	100	2	^73^
		MHV	Pasteurized wastewater	25	100	1	^31^
		MHV	Lake water, Reagent-grade water	25	100	2	^21^
		MHV	Stainless steel	4, 20, 40	20, 50, 80	8	^20^
	*Sarbecovirus*	MERS	Liquid culture medium	56, 65	100	2	^16^
		MERS	Plastic, Stainless steel	20, 30	30, 40, 80	6	^23^
		SARS-CoV-1	Plastic	28, 33, 38	95	7	^24^
			Dryed Plastic	23.5	45		
			Liquid culture medium	23.5	100		
		SARS-CoV-1	Glass, Liquid culture medium	4, 22.5, 30, 37	63, 100	8	^45^
		SARS-CoV-1	Liquid culture medium	56, 65	100	2	^17^
		SARS-CoV-1	Liquid culture medium	56	100	1	^36^
		SARS-CoV-1	Biological specimen	4	100	9	^25^
			Biological specimen, Liquid culture medium	20	100		
		SARS-CoV-1	Glass, Liquid culture medium	22	17.5	7	^29^
				58, 68	100		
		SARS-CoV-1	Aerosol, Cardboard, Copper, Plastic, Stainless steel	22	40	5	^13^
		SARS-CoV-1	Liquid culture medium, Plastic	23, 56	100	6	^18^

*median values were considered if temperatures and relative humidity were given by ranges.

Abbreviations: BCoV: Bovine coronavirus; CCV: Canine coronavirus; FCoV: Feline coronavirus; FIPV: Feline infectious peritonitis virus; HCoV: Human coronavirus; MHV: Mouse hepatitis virus; MERS: Middle East respiratory syndrome coronavirus; PEDV: Porcine epidemic diarrhea virus; PRCV: Porcine respiratory coronavirus; SARS-CoV: Severe acute respiratory syndrome coronavirus; TGEV: Transmissible gastroenteritis virus.

Several information associated with the above kinetics were gathered in an Excel spreadsheet (more details in the Data records section). For each identified kinetics, we assigned a unique kinetics key. The virus Strains, Species, Subgenus and Genus were indicated for each kinetics, as well as other conditions such as the nature of the materials (stainless steel, plastic, paper,…), the medium (liquid media, aerosol, porous and non-porous surfaces), temperature and relative humidity (expressed in range and or median value), pH (if available) etc. Other information was also indicated for each kinetics such as the initial virus load, the type of cell used for infectious viral titration (Vero, etc.) or the number of experimental replicates. Table 2 provides an overview of the extracted kinetics from SARS-CoV-2 studies, the Table 3 from studies for other coronaviruses.

### Data extraction

The persistence kinetics data were taken from texts, tables or figures in the research papers identified above. Data from tables or texts were manually filled into the database. Raw data from figures were extracted using the R package metadigitize^78^. This tool provided the possibility to process simultaneously many figures, as well as reproducibility (e.g., correcting the digitalized data, sharing digitalization). For reproducibility purposes, all files associated with raw digitalizations of our study are provided and detailed in the Data records section. The monitored time points extracted from all studies (extractions from tables or figures digitalization) were converted and expressed in hours in order to homogenize for comparative purposes between studies.

### Viral infectivity reduction parameter

The third step aimed to estimate a parameter to characterize the viral infectivity reduction for each kinetics (condition). This parameter, denoted *D* and expressed in hour, characterized the decimal log reduction time and was estimated by fitting a primary inactivation model on the extracted data^79^. The value of *D* corresponding to the inverse of the slope from a linear model^10,71^ was written as follows:

$${\log }_{10}\frac{N}{{N}_{0}}=-\frac{t}{D},$$

with *N*_0_ and *N* corresponding to the number of infectious viruses at the initial time point and the time point *t* (expressed in hour), respectively. The model was fitted independently on each kinetics using the function *nls()* from the R package nlstools^80^ running with the ‘nl2sol’ algorithm from the Port library^81^. The starting parameter values necessary for the fitting algorithm was set depending on the kinetics curves or optimized using the R package *nls2*^82^ in some rare cases of non-convergence. For each kinetics, a value of *D* was estimated as well as its uncertainty expressed by standard error value *SE*. The value of log_10_ *D* was computed accordingly for each kinetics. Finally, the coefficient of variation was calculated as the proportion: *CV*=*SE*/*D*.

### Evaluation of kinetics quality

In this work, kinetics data were collected from different publications in which the laboratory experiments were not conducted with the same design. These data represented then important variabilities, e.g. in terms of number of time points, replicates, etc. Therefore, criteria can be useful to evaluate and classify the quality of the collected kinetics. Indeed, the quality of raw data was susceptible to influence the statistical estimation of *D*. Criteria-based ranking approaches have been proposed in some predictive microbiology studies aiming to deal with difficulties in terms of data selection (inclusion or exclusion for modelling)^83,84^. Herein, for the establishment of a quality score by kinetics, we considered three criteria: (i) the number of the time points of the kinetics; (ii) the importance of the extracted point considering if it represented a single value or multiple measure (i.e. at least two technical replicates); and (iii) the value of the coefficient of variation (CV) of the estimated value of *D*, characterizing the fit quality of the inactivation model. For each kinetics, we attributed three scores corresponding to these three criteria and classified them into different categories. It is worth noting that ones can arbitrarily define the threshold values separating these categories as well as the given corresponding score values depending on the studies and the extracted dataset. In our work, for each kinetics, the score associated with the number of time points, denoted *s*_1_, was firstly defined as follows:

$${s}_{1}=\left\{\begin{array}{c}1\left({n}_{t}\le 3\right),\\ 2\left(4\le {n}_{t} < 6\right),\\ 3\left(6\le {n}_{t} < 8\right),\\ 4\left({n}_{t}\ge 8\right),\end{array}\right.$$

where *n*_*t*_ corresponds to the number of time points collected from the kinetics.

The score *s*_2_, based on the importance of points (‘unique’ or ‘multiple’), was defined as follows:

$${s}_{2}=\left\{\begin{array}{c}1\left(unique\right),\\ 3\left(multiple\right).\end{array}\right.$$

The score *s*_3_, based on the coefficient of variation *CV*_*i*_ of the kinetics *i*, was given as follows:

$${s}_{3}=\left\{\begin{array}{c}1\left(CV > 0.3\right),\\ 2\left(0.2\le CV < 0.3\right),\\ 3\left(0.1\le CV < 0.2\right),\\ 4\left(CV < 0.1\right);\end{array}\right.$$

or *s*_3_ = 1 for some kinetics for which standard errors and coefficients of variation could not be computed (kinetics with only two points).

Finally, a global score *S* taking into account all criteria was calculated for each kinetics:

$$S={s}_{1}\times {s}_{2}+{s}_{3}.$$

The calculated scores for all kinetics are gathered in Spreadsheets and R Data objects provided in the Data Records section.

## Data Records

### Intermediate data: Figure digitalization raw files

The digitalized source figures (*jpg* or *png* image files) were used in the second step of the collection procedure (see Fig. 1). The digitalization were carried using the R package *metaDigitise*^78^ that automatically created the directory denoted *‘caldat’* containing raw digitalization files to assure traceability and also to avoid re-doing manual digitalization at every run of the procedure. Raw digitalization files generated by *metaDigitise* were automatically renamed like their corresponding image files. All sources figures and digitalization raw files used herein are provided in a data repository^85^.

### Input and output quantitative data spreadsheet

Output spreadsheets were obtained at the end of the overall collection procedure under the Excel and CSV file formats (see Fig. 1) (*“DataRecord_OutputData.xlsx”* and *“DataRecord_OutputData.csv”*).

All information reported from publications (experimental conditions, figure sources, publication references, DOI, etc.) related to each kinetics used as input, as well as the corresponding estimated values as described in the Method section (*D*, coefficient de variation, scores, etc.) are present^85^. Each row represents a kinetics, each column is completed, when available, by qualitative and quantitative variables, as follows:

- **ID of each kinetics** (*Kinetics key)*, denoted for example: ‘*K00*1*’*, *‘K00*2*’*, etc.;
- **ID of each study** (*Study key)*;
- **Studied viruses** and their **classification**: *Genus*, *Sub-genus*, *Virus*, *Strain*;
- **Temperature** considered in the experimental design: temperatures were gathered by precise values reported from the publication if available (column *Temperature*) or by ranges (column *Temperature range*) in which case its median values were considered (e.g. 23.5 °C reported in *Temperature* for a range of 22–25 °C given in the publications);
- **Relative humidity** (columns *Relative humidity* and *Relative humidity range*) considered in the experimental design: as for temperatures, RH were reported as precise values and/or ranges*;*
- **pH values** (column *pH*) if available;
- Information related to the **matrices** sorted in three columns:
  - (i) the **studied matrices** (*Studied matrices – fully named*) in as described in publications, with some details;
  - (ii) the **standardized matrices** (column *Standardized matrices*), is a practical annotation to class similar matrices, such as liquid medium, stainless steel, etc.; and
  - (iii) the **medium** grouping the above matrices as four classes, which are “liquid media”, “porous surface”, “non-porous surface” or “aerosol” (column *Medium*);

- Information related to the kinetics **monitoring methods** including:
  - (i) the **quantification method** (column *Quantification method*) indicating the experimental techniques such as viral infectivity assays by different cell types;
  - (ii) the used **inoculum** (column *Inoculum*) and
  - (iii) the **replicate** (column *Replicate*) indicating if the monitored kinetics were extracted as unique time points or multiple ones;

- Sources of kinetics including
  - the bibliographic **references** (column *References*);
  - the **name of tables or figures** (column *Table or Figure of the study*) in the original publication where the kinetics raw data were transcribed or digitalized and
  - the corresponding **file names of these tables and figures** (column *Re-transcribed tables or digitalized figures*) provided in Data records allowing their re-use by other researchers;

- **Total number of points** (column *nb_*points) extracted from each kinetics;
- Different estimated values for all kinetics collected in the present study as described in the Method section:
  - (i) values of **D** (column *Dvalues*)
  - (ii) its **standard error** (column *Dvalues_stderr*) and
  - (iii) the **coefficient of variation** (column *Dvalues_CV*);
  - (iv) the **decimal log of D** (column *log*_*10*_*D)*

- For some kinetics and for comparison purposes, the estimated values of log_10_D previously estimated using another modelling approach^10^ (column *log*_*10*_*D_AEM*);
- The **scores** given to each kinetics, including *s*_1_, *s*_2_ and *s*_3_ as well as the global score *S* (columns *s*_*1*_*, s*_*2*_*, s*_3_ and *S*, respectively).

### Input and output as RData object

All input used and output obtained at the end of the collection workflow is also provided as a ready-to-use RData object (*DATASET.RData*)^85^. From this RData object, when opened in R/RStudio softwares, one can extract:

- the **input** and **output data spreadsheet** described above (object *DATASET*);
- **raw data** (from tables or figures) associated with the monitoring of each kinetics (measured values at each sampling time points), only the data above LOQ are recorded (object *kinetics_rawdata*);
- **regression plots** (inactivation linear model, see Method section) generated for each kinetics (object *regplot*). These regression plots were also exported as PDF files provided (*output_adjusted_kinetics*). The pattern of inactivation kinetics (increasing or decreasing) may be different depending on the unit used by the authors (e.g. log*TCID*_50_/ml, ${\rm{\log }}(\frac{{N}_{t}}{{N}_{0}})$, viral titer reduction in percentage, etc).

## Technical Validation

The technical validation focused on the figures’ digitalization step, since the latter remained a manual work that could probably vary from one user to another. In order to check the quality of the data collected by digitalization, this step was re-conducted repeatedly by three independent users for evaluating its repeatability and its reproducibility. This checking procedure was performed on a random sample of eleven kinetics among those collected, and each kinetics was digitalized three times per user. The values of the parameter *D* were afterwards estimated as described in previous sections. The comparison between the values of *D* estimated by different users was firstly done by fitting the major axis regression model on bootstrap data generated for each pair of users^86,87^. Afterwards, the Gage R&R tool from the R package SixSigma^88,89^ was used in order to identify and quantify the error parts in the estimated values of log_10 *D* due to the user repeatability as well as the between user reproducibility, respectively. The R scripts and data associated with the technical validation procedure are provided (see the ‘Code Availability’ section below).

**Fig. 3:**
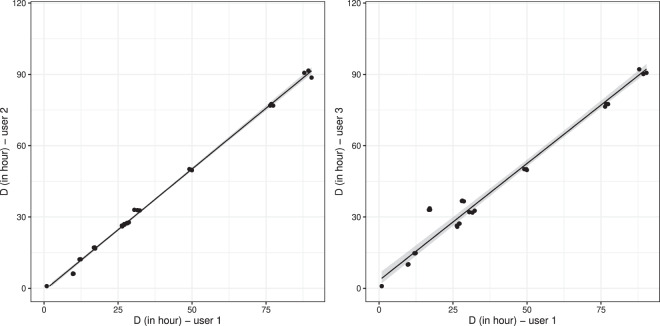
Illustration - Comparison of the D values estimated by different users performing repeatedly the figure digitalization step on the same subset of kinetics.

As illustration, the comparison between users (denoted user 1, 2 and 3, respectively) by major axis regression is plotted in Fig. 3 (user 1, plotted in the X-axis, was arbitrarily chosen herein as the reference one for comparison). The results of the Gage R&R analysis showed a good repeatability: the latter estimated a very low error part due to intra-user variation, estimated at only 0.01% of the overall variation. The error part due to the between-users variation was estimated at 1.5%. After confrontation between experimenters, this part of inter-users error can be explained by the difficulties for choosing the points to be digitalized. Indeed points below the limit of quantification (LOQ) should not be included for avoiding bias. This choice could then strongly influence the estimation of log_10_ *D* as illustrated in Fig. 3, since this parameter conditioned the slope of the linear model fitted to the chosen data points. Yet, in many articles, the information LOQ is not provided. In such conditions, it is up to scientist in charge of digitalization to include/exclude points to be included. This is prone to introduce uncertainty especially for points corresponding to the end of the experiment. In view of this user-dependent choice, in the present study, we provided then all raw digitalization files that be imported, re-used or modified by other users if needed according to their expertise.

## Usage Notes

### Re-use of figure digitalization files

The digitalization step were done using the R package *metaDigitise*^78^ providing reproducible and flexible tools for tracing every digitalization. In practice, the digitalizations were done using R commands (check the R scripts provided in the Code Availability section) allowing users to process the different image files ready-to-digitalize. This process consisted, for each image (plot), to click manually on the different chosen points of the image to calibrate the plotted axes and convert afterwards the different clicked points (from curves, barplots, etc.) to numerical values saved in R object. The different groups of points can be assigned with user-defined group names in order to separate different kinetics from the same image if necessary. For each digitalized image, a digitalization file is automatically generated in a specific directory, denoted *‘caldat’* to ensure the traceability of this manual step. Indeed, such a file can give the possibility, using R commands, to import the numerical values already digitalized and/or edit/recalibrate some values/points by other users if needed without having to re-process the whole image .

**Fig. 4:**
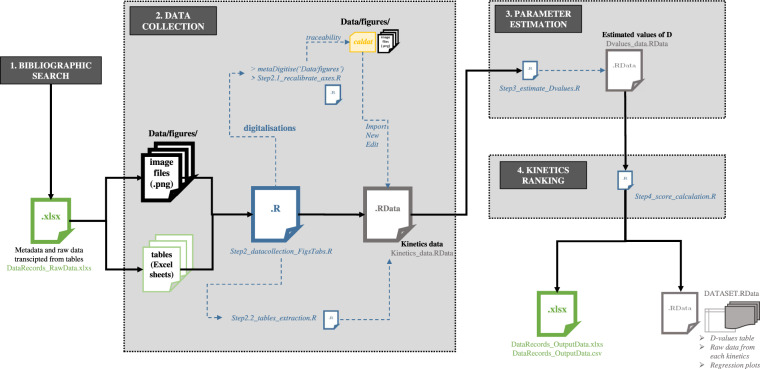
Detailed schema of the data collection procedure including the used R scripts and data records files.

## Data Availability

All data records files and R scripts used for the data collection procedure are schematized in Fig. 4 and available at online repositories^85^: https://github.com/lguillier/SACADA_Database and https://zenodo.org/record/6572948#.YouM3ajP3tR. The database (bibliographic references) was also extracted as RIS and BIB files for open-source software.
